# Reply to López-Moreno, M.; López-Gil, J.F. Comment on “Hernández-Lorca et al. Effects of Dry-Cured Ham Consumption on Cardiometabolic and Vascular Health in Adults: A Systematic Review and Meta-Analysis of Human Intervention Studies. *Foods* 2026, *15*, 1198”

**DOI:** 10.3390/foods15101807

**Published:** 2026-05-20

**Authors:** Manuel Hernández-Lorca, Desirée Victoria-Montesinos, Ana María García-Muñoz, Eva Salazar, Adela Abellán

**Affiliations:** 1 Department of Nutrition and Food Technology, Universidad Católica de Murcia-UCAM, Campus de los Jerónimos, 30107 Murcia, Spain; mhernandez2@ucam.edu (M.H.-L.); esalazar@ucam.edu (E.S.); aabellan@ucam.edu (A.A.); 2 Faculty of Pharmacy and Nutrition, UCAM Universidad Católica de Murcia, 30107 Murcia, Spain

We thank the authors of the Comment for their careful reading of our article and for raising important interpretative and methodological considerations [1]. We appreciate the opportunity to clarify the intended scope of our conclusions and to further contextualize the quantitative synthesis.

We agree with the general principle that dietary effects are inherently comparative and should be interpreted in relation to the food or dietary context being replaced. In nutrition research, the importance of asking “instead of what?” and considering the broader dietary context has been emphasized repeatedly, and we agree that making this framework more explicit can improve interpretative precision [2,3,4]. In this sense, the causal contrast addressed by a dietary intervention is not defined by the intervention food alone, but by the combination of the intervention, comparator, population, dose, duration, and outcome assessed. Therefore, the findings of our review should be interpreted as comparator-specific, short-term effects on intermediate cardiometabolic and vascular markers, rather than as evidence of an inherent or absolute cardiometabolic neutrality of dry-cured ham.

At the same time, we would like to note that our original wording was intentionally cautious. In the published article [5], we concluded that dry-cured ham consumption “does not appear to adversely affect conventional cardiometabolic risk markers in adults”, while explicitly emphasizing the limited number of trials, their short duration, the substantial between-study heterogeneity, and the need for cautious interpretation.

Regarding the choice of comparators, we would respectfully clarify that the evidence synthesized in our review was not restricted exclusively to comparisons between dry-cured ham and cooked ham. Although two randomized crossover trials used cooked ham as the comparator [6,7], the broader qualitative synthesis also included additional comparator contexts, including a diet without Iberian cured ham and pre–post comparisons against habitual or basal diet [8,9,10].

Therefore, the strongest causal interpretation derived from the randomized evidence is that replacing cooked ham with dry-cured ham did not worsen the cardiometabolic markers assessed within the duration tested. This interpretation is narrower than a general statement of cardiometabolic neutrality and does not imply that dry-cured ham would have the same effect if compared with minimally processed animal protein sources, plant-based alternatives, or dietary patterns specifically designed for cardiovascular prevention. This distinction is relevant because substitution analyses suggest that replacing red or processed meat with plant-based foods such as nuts, legumes, or whole grains may be associated with more favourable cardiometabolic and mortality outcomes [11]. The additional non-randomized and pre–post studies should therefore be considered complementary and hypothesis-generating, as they provide a weaker basis for causal inference than randomized controlled comparisons.

Accordingly, we agree that our findings should not be interpreted as evidence of intrinsic “healthfulness” or neutrality in absolute terms, nor as grounds to override the broader observational and public-health literature on processed meat intake. Large prospective cohorts and evidence syntheses have reported associations between higher processed meat consumption and adverse cardiometabolic outcomes, including cardiovascular disease, type 2 diabetes, and all-cause mortality [12,13,14,15]. Our intention was instead to synthesize the currently available short-term human intervention evidence on a specific product with a distinct food matrix and nutritional profile, while carefully acknowledging the limitations of that evidence. Indeed, in the original Discussion, we explicitly recognized the potential replacement effect, noting that in several studies dry-cured ham was introduced within the habitual diet or substituted for other meat products, and that its apparent health impact may therefore partly depend on the nutritional profile of the food it replaces [5].

Thus, the issue raised by the Comment does not invalidate the synthesis itself but rather helps define more precisely the level at which its conclusions should be interpreted. Our original review addressed whether the available intervention evidence suggested adverse changes in conventional cardiometabolic and vascular markers after dry-cured ham consumption. This distinction is essential because dietary recommendations are ultimately based not only on the effect of adding a food, but also on the food that it replaces within the overall diet.

With respect to the quantitative synthesis, the small number of randomized trials limits the precision and certainty of the pooled estimates. However, the existence of only a few studies does not automatically preclude quantitative synthesis, provided that uncertainty is clearly acknowledged and conclusions remain appropriately restrained [16,17]. This was the rationale for our approach. In our review, random-effects models were used to account for between-study heterogeneity, and outcomes not considered suitable for pooling were synthesized narratively [5].

We acknowledge that, when only a small number of studies is available, the estimation of between-study variance is inherently imprecise and pooled estimates may be sensitive to modelling assumptions [16,17]. For this reason, the meta-analysis was not intended to provide definitive evidence of a single generalizable effect, but rather a descriptive quantitative summary of the limited randomized evidence available for sufficiently comparable outcomes. In this setting, its value lies less in producing a definitive pooled estimate and more in transparently summarizing the direction, magnitude, uncertainty, and consistency of the available evidence.

We likewise acknowledge that crossover trials require analytical assumptions when within-subject correlation coefficients are not reported. This is a recognized challenge in evidence synthesis rather than a methodological irregularity specific to our review. Current methodological guidance indicates that non-standard designs such as crossover trials should be analyzed using methods appropriate to the design and that, when study authors do not account for correlations arising from the design, approximate methods can often be applied by review authors [18]. In line with this guidance, we stated the assumed within-subject correlation coefficient in the Methods section and conducted sensitivity analyses using an alternative plausible value. Importantly, these sensitivity analyses did not materially alter the direction or statistical interpretation of the pooled estimates for lipid parameters, blood pressure, or fasting blood glucose [5]. Therefore, while we fully agree that the precision of the pooled estimates should be interpreted cautiously, the overall pattern of findings does not appear to have been driven solely by the selected correlation assumption.

Concerning heterogeneity, the very high I^2^ values observed across several outcomes warrant caution. At the same time, I^2^ should not be interpreted as an absolute indicator that a meta-analysis is inherently uninformative or invalid. As emphasized by Borenstein and colleagues, heterogeneity statistics must be interpreted in conjunction with effect magnitudes, clinical and methodological diversity, and the purpose of the synthesis [19]. In our case, the observed heterogeneity likely reflects clinically relevant differences across studies, including participant characteristics, baseline cardiometabolic risk, ham type, dose, intervention duration, comparator product, and study design [5,6,7,8,9,10]. Therefore, the pooled estimates should not be interpreted as representing an interchangeable effect across all dietary contexts. Instead, they should be read together with the structured qualitative synthesis and with the limitations already described in the original article, particularly the limited number of trials, their short duration, the use of intermediate biomarkers, and the potential influence of replacement effects [5].

Taken together, the most cautious interpretation is that the randomized evidence supports a narrow, comparator-specific conclusion, whereas the broader intervention evidence provides complementary but less definitive support. Both interpretations are comparator-dependent and should not be extrapolated to long-term disease outcomes, broader processed meat categories, or substitutions with minimally processed or plant-based alternatives.

Overall, we appreciate the Comment authors’ emphasis on comparator interpretation and limits of inference. The findings are best understood within the comparator contexts and short-term intervention settings actually studied. In our view, this interpretation refines, rather than revises, the cautious wording already used in the original article. Accordingly, the available short-term intervention evidence suggests that dry-cured ham consumption does not appear to adversely affect conventional cardiometabolic risk markers under the specific study conditions assessed. As noted, the most robust inference supported by the randomized trials is that replacing cooked ham with dry-cured ham did not worsen the markers evaluated in the short term. These findings should not be extrapolated to long-term disease outcomes, broader processed meat categories, or replacement scenarios that were not directly tested.

## Data Availability

The original contributions presented in this study are included in the article. Further inquiries can be directed to the corresponding authors.
